# Supplementary material for: Enhanced ε-Poly-L-Lysine Production in Streptomyces albulus through Multi-Omics-Guided Metabolic Engineering
Source: Biomolecules. 2024 Jun 25;14(7):752. doi: 10.3390/biom14070752 (PMC11274744; doi:10.3390/biom14070752)
Supplement: Supplementary file 1 [file biomolecules-14-00752-s001.zip › biomolecules-3034252-supplementary.pdf]

Supplementary Materials

Enhanced ε-Poly-L-Lysine Production in *Streptomyces albulus* through Multi-Omics-Guided Metabolic Engineering

Liang Wang †, Hao Yang †, Mengping Wu, Hongjian Zhang, Jianhua Zhang and Xusheng Chen *

Key Laboratory of Industrial Biotechnology, School of Biotechnology, Jiangnan University, Ministry of
Education, Wuxi 214122, China; wangl@jiangnan.edu.cn (L.W.); 7220201025@stu.jiangnan.edu.cn (M.W.); jhzhang@jiangnan.edu.cn (J.Z.)
* Correspondence: chenxs@jiangnan.edu.cn; Tel./Fax: +86-510-85918296
† These authors contributed equally to this work.

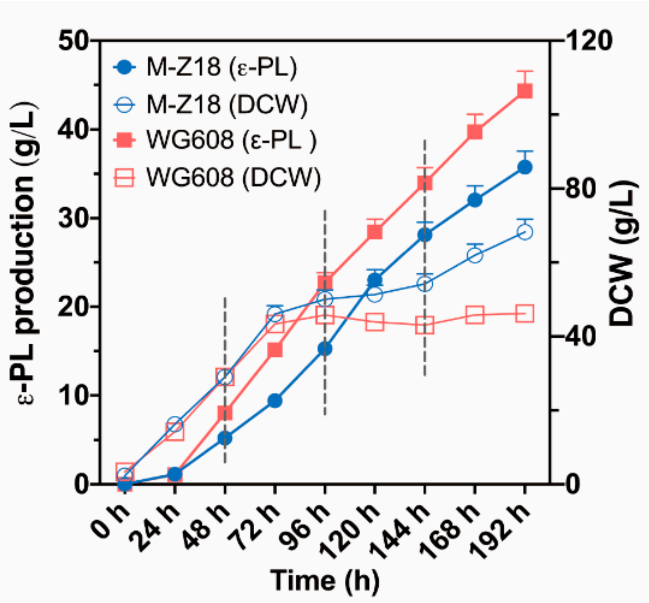

Figure S1: Fermentation performances of M-Z18 and WG608 during the fed-batch fermentation. The blue circle represents strain M-Z18; the red square represents strain M-Z18; the solid circle/square represents ε-PL production; the empty circle/square represents cell dry weight (DCW).

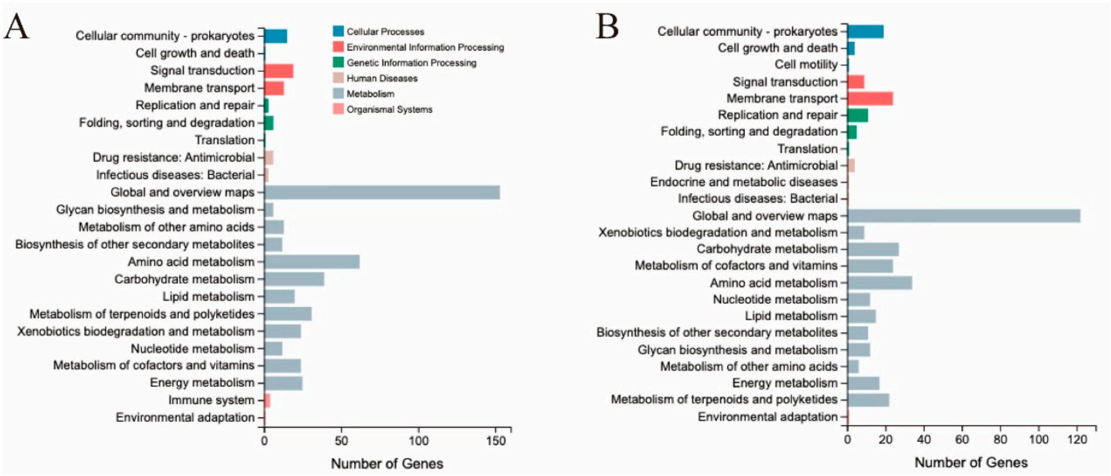

Figure S2: KEGG pathway classification of the upregulated (A) and downregulated (B) differential expressed genes during the whole ε-PL fermentation process.

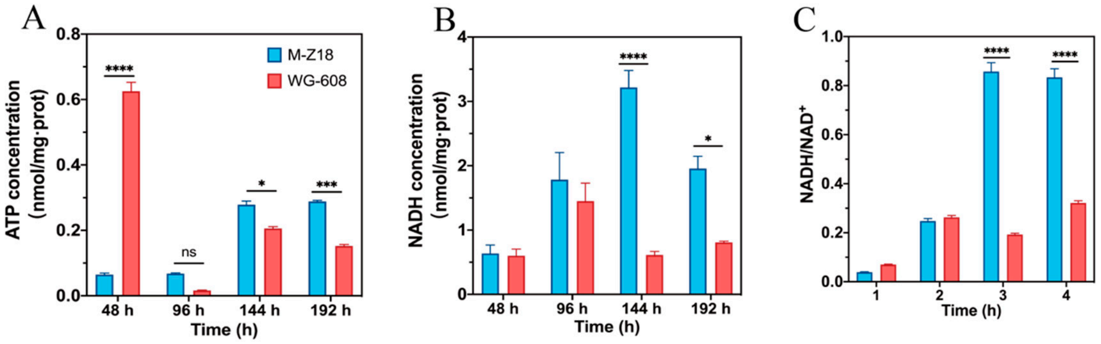

Figure S3: Differences in intracellular ATP concentration (A), NADH concentration (B), and NAD/NAD$^+$ ratio (C) between M-Z18 and WG608. The statistical analysis was performed by one-way ANOVA analysis; *, *** and **** indicate $p \leqslant 0.05$, $p \leqslant 0.001$ and $p \leqslant 0.0001$ relative to the control (WG608), respectively.

Table S1: Primers used in this study.

Primers	Sequence (5'-3')	Description
ppk1-F	ggttggtaggatccacatatgCGTGGTCGTCCGCAACC	Single gene overexpression DNA fragment cloning
ppk1-R	ggttggtaggatccacatatgCGGTCGTCGTCCGCAACC	
C-*ppk2B*^{cg}-F	ggttggtaggatccacatatgATTGGCTAAAATCCACAGCCTT	
C-*ppk2B*^{cg}-R	atccaaagacgcCTAGTCACCGATCTGGTCGCG	
C-2-*pap*-F	gtgactagGCGTCTTTGGATATTTGTTGTCTTA	
C-2-*pap*-R	ctatgacatgattacgaattcTTAATCGGTATCTCGATCAGCTTTT	
C-3-*pap*-F	gtgactagGCGTCTTTGGATATTTGTTGTCTTA	
C-3-*pap*-R	acgacgaccgTTAATCGGTATCTCGATCAGCTTTT	
C-*ppk1*-F	taccgattaaATGCGTGGTCGTCCGCAACC	
C-*ppk1*-R	ctatgacatgattacgaattcTCAACGTGGCAGCCGGACTCC	
zwf-F	ggttggtaggatccacatatgGCCGTCGGGCCTGGTCAT	
zwf-R	ctatgacatgattacgaattcTCATGGCCGACGCCAGCT	
A-F	ggttggtaggatccacatatgATGGACATCGGGATCTCCTGG	Gene co-overexpression DNA fragment cloning
A-R	aagaagcgcgggtccgTCATGGCAGAAGCGAGGACG	
S-F	atgaCGGACCCGCGCTTCTTCT	
S-R	atgttggtcatCGAGCACCACCTCGCCCT	
D-F	tggtgctcgATGACCAACATCCGCGTAGC	
D-R	catgattacgaattcgatatcTTAGACGTCGCGTGCGATC	
pls-F	cgagataccgattaagaattcTCTAAGTAAGGAGTGTCCA ATGTCGTCGCCCCTTCTCG	
pls-R	ctatgacatgattacgaattcACTGCATCGGGTCACGCG	
PE-F	aatcatgtGCCAAGCTTGGGCTGCAG	
PE-R	tagccaatcatatgtggatccTACCAACCGGCACGATTGTG	
ASD-F	tcgtgccggttggtaggatccATGGACATCGGGATCTCCTGG	
ASD-R	cccaagcttggcACATGATTACGAATTCGATATCTTAGACG	
ddh^{bs} -F	CATATGTCCGCCATCCGC	Verify primers
ddh^{bs} -R	ACTTCGGGGACAGCAGGC	
ddh^{cg} -F	CATATGACCAACATCCGCGTC	
ddh^{cg} -R	ATCAGGTCGTCCAGGTTCTCC	
pIB-F	ggttggtaggatccacatatgCGGTCGTCGTCCGCAACC	
pIB-R	GCACGACAGGTTTCCCGACTG	
2-F	AGTGGCAGAACTGGACCAAGGA	
2-R	CTCATCGCATCGGCAACCTGTT	
3-F	AGTGGCAGAACTGGACCAAGGA	
3-R	GATGCTGTGGATGCGGAAGAGG	
Y_{ASD}-F	GCCTCTTCGCTATTACGCCAG	
Y_{ASD}-R	GACCAGATGCTCGCCTACAACT	
Y_{PE}-F	GGCTACGAGGTCGAGGTCAACT	
Y_{PE}-R	ATGGCTTCTCCAACGCAACAGT	

Table S2: Differential expression metabolites between *S. albulus* WG608 and *S. albulus* M-Z18.

Pathway	Metabolites	KEGG ID	Fold change (log2)		
			48h	96h	144h
EMP	Glucose-6P	C00092	0.56	0.67	0.93
	Glyceraldehyde-3P	C00597	1.27	1.06	1.47
	Phosphoenolpyruvate	C00074	1.51	1.21	1.98
	pyruvate	C00022	0.37	0.28	1.14
PPP	Ribulose-5P	C00117	6.51	7.64	12.91
	sedroheptulose-7P	C05382	0.82	1.25	1.10
TCA	citric	C00158	1.27	0.96	6.18
DAP	D-aspartate	C00402	2.16	2.07	1.62
	L-asparagine	C00152	0.65		1.95
	L-lysine	C00047	1.40	1.24	1.28
	(r)-homocitric acid	C01251	0.91	0.78	1.45
	L-saccharopine	C00449	0.31	3.44	1.63
	L-threonine	C00188	1.64	1.68	
Histidine metabolism	L-histidine	C00135	2.98	3.23	1.73
	L-glutamic acid	C00025	0.42	0.41	1.27
	N.pi.-methyl-l-histidine	C01152	0.25	0.06	0.40
	Carnosine	C00386	23.50	12.53	4.17
Glycine, serine and threonine metabolism	Betaine	C00719	0.12	9.20	0.45
	Glycine	C00037	1.90	1.43	
	Creatine	C00300	1.14	0.18	1.81
	L-allo-threonine	C05519	2.13	1.54	
	L-serine	C00065	2.15	1.70	
	L-threonine	C00188	1.64	1.45	
	Choline	C00114	0.48	0.41	0.69
	L-tryptophan	C00078	6.75	2.69	1.65
	L-serine o-phosphate	C01005	1.08	3.05	4.01
	Pyruvic acid	C00022	0.37	0.28	
	dihydroxypropanoic acid	C00258	1.35	0.40	0.79
	2-oxobutyric acid	C00109	0.44	0.27	1.23
Phenylalanine, tyrosine and tryptophan biosynthesis	Indole	C00463	4.13	2.65	1.32
	(-)-shikimic acid	C00493			
	L-phenylalanine	C00079	2.45		1.22
	L-tryptophan	C00078	6.75	2.69	1.35
	Phosphoenolpyruvic acid	C00074	1.51	1.21	1.98
	L-tyrosine	C00082	4.72	3.04	1.50
	Phenylpyruvic acid	C00166	6.78	9.75	1.76

Cysteine and methionine metabolism	L-serine	C00065	2.63	1.91	
	Acpc	C01234	0.66	0.53	
	5'-methylthioadenosine	C00170	0.38	0.58	
	1,2-dihydroxy-3-keto-5-methylthiopentene	C15606	0.15	0.18	0.39
	Sulfuric acid	C00059	0.37	0.34	0.55
	2-oxobutyric acid	C00109	0.44	0.27	1.23
	L-serine	C00065	2.15	1.91	
	Pyruvic acid	C00022	0.37	0.28	
	L-serine o-phosphate	C01005		3.05	4.01
Valine, leucine and isoleucine	Valine	C00183		1.45	
	Acetoacetate	C00164	0.30	0.26	0.73
	Leucine	C00123	3.62	2.38	1.47
	Alpha-ketoisovaleric acid	C00141	0.05	0.01	0.04
	L-threonine	C00188	0.68	1.45	
	2-isopropylmaleic acid	C02631	1.27	1.47	1.80
Arginine and proline metabolism	Spermidine	C00315	0.12	0.63	1.43
	L-arginine	C00062	1248.50	235.72	36.88
	4-acetamidobutyric acid	C02946	0.21	0.29	0.73
	Creatine	C00300		0.18	1.81
	Gamma-aminobutyric acid	C00334	1.42	1.35	0.77
	L-glutamic acid	C00025	0.42	0.38	1.27
	N-methylhydantoin	C02565	0.50	0.15	0.52
	Pyruvic acid	C00022	0.37	0.28	1.14
	Gamma-aminobutyric acid	C00334	0.44	0.37	0.77
	4-oxoproline	C01877		0.51	1.38
	L-proline	C00148	2.32	2.16	1.25
	5-aminovaleric acid	C00431	3.51	4.27	1.44
	3-hydroxy-l-proline	C05147	1.30	9.80	8.39
Alanine, aspartate and glutamate metabolism	γ-amionbutyric acid	C00334	1.42	0.37	0.77
	L-glutamine	C00064	2.42	0.79	
	D-aspartate	C00402	2.16	2.07	1.62
	L-glutamic acid	C00025	0.42	0.38	1.27
	L-asparagine	C00152	0.65		1.95
	Succinate	C00042	0.21	0.41	0.59
	Citrate	C00158	1.27		6.18
	Pyruvic acid	C00022	0.37	0.28	1.14
